# Oral Pathology and Therapeutics

**Published:** 1905-05

**Authors:** 


### Bibliography.

ORAL PATHOLOGY AND THERAPEUTICS. A Systematic Presentation of the Subject from the Stand-Point of Modern Therapeutics. With One Hundred and Sixteen Illustrations. By ELGIN MAWHINNEY, D.D.S., Chicago, Professor of Special Pathology, Materia Medica, and Therapeutics. Northwestern University Dental School, etc. The Consolidated Dental Manufacturing Company, New York, N. Y.; Claudius Ash & Sons (Limited), London, England, 1905.

The author says in the Preface, "This volume is presented with the hope that it may furnish at least a rational scientific basis for the management of many oral diseases." The reader will naturally conclude from this that the book will satisfy all reasonable demands, and will prove a satisfactory arrangement of dental pathological subjects, embracing etiology and treatment. That this is not accomplished on its pages will be made evident as the reader proceeds with his examination.

The author seems to deem any leading up to the main subject as unnecessary; and he therefore opens at once in Chapter I. with "Dental Caries." The history of this important subject is confined to just one-half page, and to that extent fails to be even an outline of the work of the past two centuries. He then follows this meagre sketch with "Recent Theories," and devotes exactly *five lines* to Miller. In view of the fact that the world of dentistry recognizes Miller as having given not only the most thorough scientific elucidation of the subject, but the final one in regard to the origin of this disease, this unsatisfactory paragraph leaves a very bad impression on the mind of the reader.

This very objectionable method of treating important matters, not apparently interesting to the author, seems to be part of the plan of the volume, for it is found, to a greater or less extent, in the majority of the chapters.

Chapter III. seems to have been taken without revision from one of the author's lectures, and even for that its style might be greatly improved for the benefit of the rising dental generation.

Quotation from a portion of two pages will best illustrate this, leaving the reader to draw his own conclusions. "When you get into practice here are some of the things you must consider. First, the personnel of the dentist. You must have a professional air about you. You want to recognize the fact that our calling is something more than trade; that it is a profession; there is a dignity about it, a professional air. Kind in manner and speech, and, as some one has said, 'Keep your voice low!' There is a whole lot in that." This may be all very good advice to a body of careless students, but wholly inappropriate, as well as that which follows, in a work on dental pathology.

The intelligent reader, after trying to peruse these pages, will be inclined to lay down the book and let it pass out of sight as a mass of words in exceeding bad taste, but this hasty opinion would hardly do the author full justice, for there are some good things stored within its pages.

The following is the author's idea, in part, at least, of "Systemic Medication:" "Morphine is one of the alkaloids of opium, and the one most used. For purposes of alleviating pain it is without a rival. It is given in doses of one-eighth grain an hour before the operation and another fifteen minutes before. I never tell my patients what they are taking when they take morphine, because so many have a prejudice against it."

The following will interest the critical reader. The author has described a case of "Calcific Degeneration of the Pulp," and then proceeds: "Well, he was an individual who wouldn't bear much work with his teeth. He was one of those individuals who didn't believe much in having much done with his teeth, anyway, and consequently had neglected them until he had lost a number of his molars. He decided on his own hook when one couldn't find the cause of the trouble that he would have a certain one out." The following statement seems even remarkable for Chicago: "In all acute suppuration we have a decided rise in temperature, as I have stated, 101, 102, 103, 104, 105, or even 106 occasionally from an acute alveolar abscess." While death has resulted from an alveolar abscess, it is exceedingly rare, and the ordinary abscess of the peridental membrane will rarely raise the temperature above 103°.

Except in certain special cases, where he is very profuse in his credits, he does not seem to consider this trifling courtesy of much moment. In his usual breezy way he will take up one method and give his ideas with a freedom peculiarly characteristic. In the chapter devoted to bleaching discolored teeth this is prominent. Credit is, of course, not given to the author of the chlorine method, nor is this described properly.

On page 166 the author classes the reviewer with those who entertain the theory that the so-called serumal calculus is the "result of the presence in the system of unusual quantities of urates and uric acid." If he will kindly drop the name of Truman from the list in future editions, it will be appreciated, as the owner of the name mentioned entertains quite opposite views.

There are some good common-sense ideas expressed on the pages of this book, and in one chapter, on "Infection, Instrument Sterilization, and Germicides," the work is so much superior to the rest of the production that it is difficult to realize it is from the same pen.

The book will not add much to our knowledge of pathological conditions, and in its present form is not altogether creditible to the judgment of the author; but if he will eliminate in the next edition much of the *talk* and confine it to practical teaching, he may make a book worthy the study of the practitioner and student.

